# TEP or TAPP for recurrent inguinal hernia repair—register-based comparison of the outcome

**DOI:** 10.1007/s00464-017-5416-1

**Published:** 2017-02-03

**Authors:** F. Köckerling, R. Bittner, A. Kuthe, M. Hukauf, F. Mayer, R. Fortelny, C. Schug-Pass

**Affiliations:** 1 Department of Surgery and Center for Minimally Invasive Surgery, Academic Teaching Hospital of Charité Medical School, Vivantes Hospital, Neue Bergstrasse 6, D-13585 Berlin, Germany; 2 Winghofer Medicum, Hernia Center, Winghofer Strasse 42, D-72108 Rottenburg am Neckar, Germany; 3 Department of General and Visceral Surgery, German Red Cross Hospital, Lützerodestrasse 1, D-30161 Hanover, Germany; 4 StatConsult GmbH,, Halberstädter Strasse 40 a, D-39112 Magdeburg, Germany; 5 Department of Surgery, Paracelsus Medical University, Müllner Hauptstrasse 48, 5020 Salzburg, Austria; 6 Department of General-, Visceral- and Oncologic Surgery, Wilhelminen Hospital, Montleartstrasse 37, 1160 Vienna, Austria

**Keywords:** Hernia, Recurrent inguinal hernia, TEP, TAPP, Seroma

## Abstract

**Introduction:**

The guidelines of the international hernia societies recommend laparo-endoscopic inguinal hernia repair for recurrent hernias after open primary repair. To date, no randomized trials have been conducted to compare the TEP vs TAPP outcome for recurrent inguinal hernia repair. A Swiss registry study identified only minor differences between the two techniques, thus suggesting the equivalence of the two procedures.

**Materials and Methods:**

Between September 1, 2009 and August 31, 2013 data were entered into the Herniamed Registry on a total of 2246 patients with recurrent inguinal hernia repair following previous open primary operation in either TAPP (*n* = 1,464) or TEP technique (*n* = 782).

**Results:**

Univariable and multivariable analysis did not find any significant difference between TEP and TAPP with regard to the intraoperative complications, complication-related reoperations, re-recurrences, pain at rest, pain on exertion, or chronic pain requiring treatment. The only difference identified was a significantly higher postoperative seroma rate after TAPP, which was influenced by the surgical technique, previous open primary operation and EHS-classification medial and responded to conservative treatment.

**Conclusion:**

TEP and TAPP are equivalent surgical techniques for recurrent inguinal hernia repair following previous open primary operation. The choice of technique should be tailored to the surgeon’s expertise.

In the updated guidelines on laparoscopic (TAPP) and endoscopic (TEP) treatment of inguinal hernia, the International Endohernia Society states as grade A recommendation for primary inguinal hernias, following comparison of the two laparo-endoscopic techniques, that both techniques are acceptable treatment options for inguinal hernia repair and there are sufficient data to conclude that both TAPP and TEP are effective methods of laparo-endoscopic primary inguinal hernia repair (1). A comparative Swiss registry study with a large proportion of TEP operations identified for primary inguinal hernias higher perioperative complication rates for TEP (2), whereas a German registry study with a large proportion of TAPP operations detected a higher perioperative complication rate for TAPP (3). Comparison of the outcome of laparo-endoscopic inguinal hernia repair for primary inguinal hernias with that of recurrent inguinal hernias revealed that recurrent procedures are associated with significantly higher postoperative complication rates, complication-related reoperation rates, and higher pain and recurrence rates (4).

There are six meta-analyses available for comparison of laparo-endoscopic with open repair of recurrent inguinal hernias (5, 6, 7, 8, 9, 10). The meta-analysis by Pisanu (8) contained the largest number of exclusively prospective randomized trials (RCTs) (11, 12, 13, 14, 15, 16, 17). All the RCTs included in that meta-analysis compared the laparo-endoscopic procedures with the open Lichtenstein technique. Three RCTs compared the Lichtenstein operation with TAPP (13, 15, 17), two RCTs compared it with TEP (11, 16), and two RCTs compared it with both TEP and TAPP (12, 14).

In the meta-analysis, only joint comparison of the two laparo-endoscopic techniques (TEP, TAPP) with the open Lichtenstein technique was performed. There was no high risk of bias in any of the included trials (8). The meta-analysis by Pisanu et al. (8) detected for laparo-endoscopic repair of recurrent hernias a significantly lower chronic pain rate and significantly earlier resumption of normal everyday activities. Compared with the Lichtenstein operation, the operative time for laparo-endoscopic procedures was significantly longer (8). On the basis of the meta-analyses, the European Hernia Society recommends laparo-endoscopic inguinal hernia repair for recurrent hernias after conventional open repair (10, 18). No distinction is made here between the laparo-endoscopic TEP and TAPP techniques.

To date, no randomized trials have been conducted to compare the TEP vs TAPP outcome for recurrent inguinal hernia repair following previous open repair. A Swiss registry study (19) compared the outcome of a total of 1309 laparo-endoscopic recurrent operations, of which 1022 used the TEP technique and 287 the TAPP technique. A significantly higher intraoperative complication rate and longer operative time was identified for the TEP group. The postoperative length of hospital stay was longer for patients undergoing TAPP (19). Surgical postoperative complications, general postoperative complications, and conversion rates were not significantly different (19). The authors concluded that the absolute outcome differences are small and that both techniques appear to be safe and effective for patients undergoing laparo-endoscopic repair for unilateral recurrent inguinal hernia (19).

Based on data from the Herniamed Registry (20), the present analysis now compares the outcome of elective laparo-endoscopic recurrent unilateral inguinal hernia repair in men following previous open operation.

## Patients and methods

The Herniamed Registry is a multicenter, internet-based hernia registry (20) into which 427 participating hospitals and surgeons engaged in private practice (Herniamed Study Group) have entered data prospectively on their patients who had undergone hernia surgery. All postoperative complications occurring up to 30 days after surgery are recorded. On one-year follow-up, postoperative complications are once again reviewed when the general practitioner and patient complete a questionnaire. They are also asked about any re-recurrence, pain at rest, and on exertion as well as pain requiring treatment. This present analysis compares the prospective data collected for all male patients with a minimum age of 16 years who had undergone elective recurrent unilateral inguinal hernia repair using either transabdominal preperitoneal patch plasty (TAPP) or total extraperitoneal patch plasty (TEP).

**Fig. 1:**
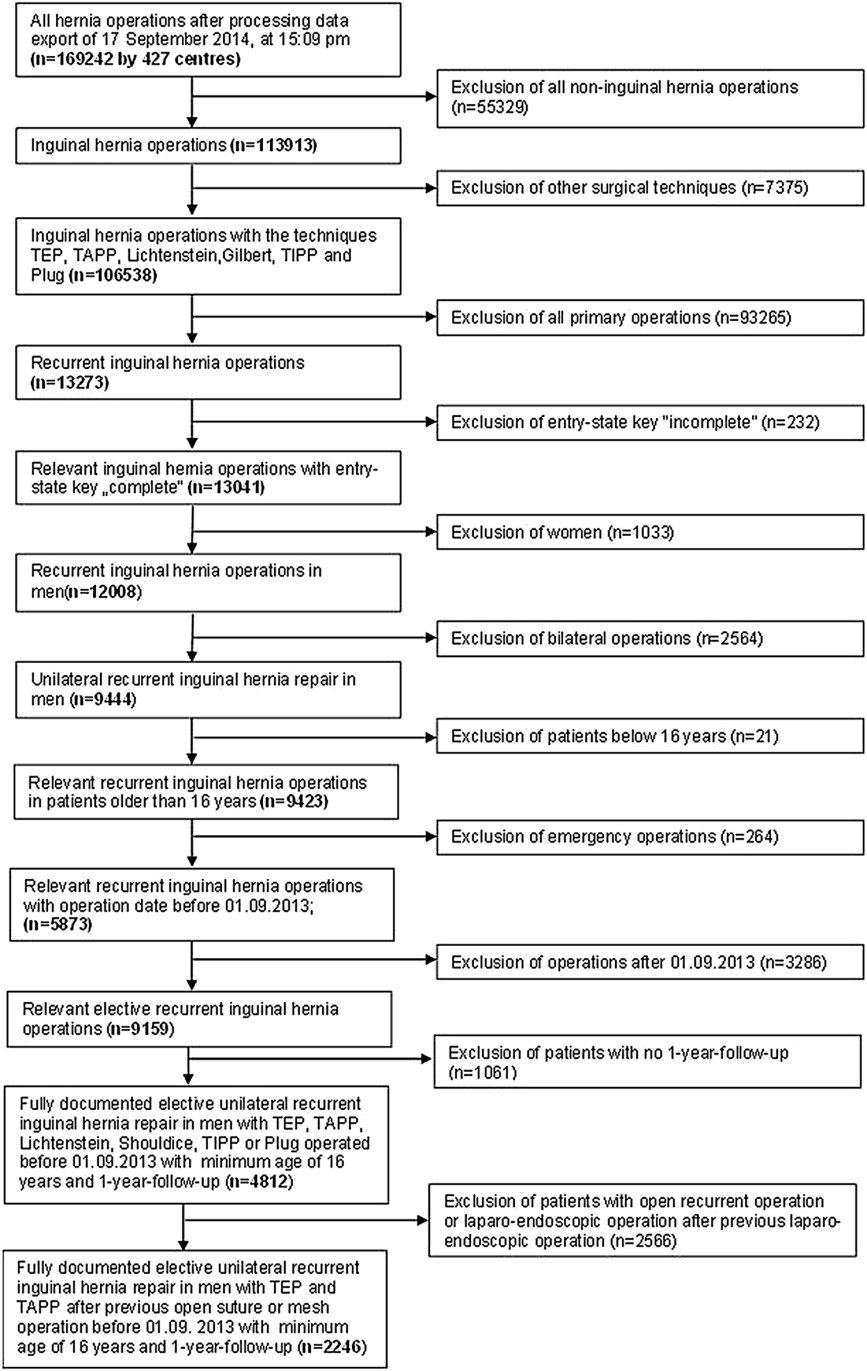
Flowchart of patient inclusion

In total, 2,246 patients were enrolled between September 1, 2009, and August 31, 2013 (Fig. 1). Of these patients, 782 (34.8%) had TEP and 1,464 (65.2%) TAPP repair. All the patients had to have one-year follow-up data available (follow-up-rate: 100%).

The majority of primary unilateral repairs in the Herniamed Registry have been performed as open mesh repair (Lichtenstein) with 32.5% and laparo-endoscopic mesh repair (TAPP, TEP) with 49.9%. Non-mesh repairs (Shouldice, Bassini) in primary unilateral inguinal hernia repair in the Herniamed Registry make only a contribution of 4.7% of all cases.

The demographic and surgery-related parameters included age (years), BMI (kg/m^2^), ASA-score (I, II, III, IV) as well as EHS classification (hernia type: medial, lateral, femoral, scrotal) and defect size: grade I = < 1.5 cm, grade II: 1.5–3 cm, grade III: > 3 cm) (21) and general risk factors (nicotine, COPD, diabetes, cortisone, immunosuppression, etc.). Risk factors were dichotomized, i.e., ‘yes’ if at least one risk factor is positive and ‘no’ otherwise.

The dependent variables were intra- and postoperative complication rates, number of reoperations due to complications as well as the one-year results (re-recurrence rate, pain at rest, pain on exertion, and pain requiring treatment).

All analyses were performed with the software SAS 9.2 (SAS institute Inc. Cary, NY, USA) and intentionally calculated to a full significance level of 5%, i.e., they were not corrected in respect of multiple tests, and each* p* value ≤ 0.05 represents a significant result. To discern differences between the groups in unadjusted analyses, Fisher’s exact test was used for categorical outcome variables, and the robust *t* test (Satterthwaite) for continuous variables.

To rule out any confounding of data caused by different patient characteristics, the results of unadjusted analyses were verified via multivariable analyses in which, in addition to TEP or TAPP operation, other influence parameters were simultaneously reviewed.

To identify influence factors in multivariable analyses, the binary logistic regression model for dichotomous outcome variables was used. Estimates for odds ratio (OR) and the corresponding 95% confidence interval based on the Wald test were given. For influence variables with more than two categories, one of the latter forms was used in each case as reference category. For age (years) the 10-year OR estimate and for BMI (kg/m^2^) the 5-point OR estimate were given. Results were presented in tabular form, sorted by descending impact.

## Results

### Univariable analysis

In the TEP group, recurrent repair was performed for *n* = 554/782 (70.8%) patients following previous suture repair and for *n* = 228/782 (29.2%) after mesh repair. In the TAPP group, recurrent repair was performed for *n* = 974/1,464 (66.5%) patients after suture and *n* = 490/1,464 (33.5%) after mesh repair (Table 1).

**Table 1:** Laparo-endoscopic recurrent unilateral inguinal hernia repair in men and previous operation

	Previous operation				Total	
	Suture		Open mesh			
	N	%	N	%	N	%
Procedure	554	36.3	228	31.8	782	34.8
TEP						
TAPP	974	63.7	490	68.2	1464	65.2
Total	1528	100.0	718	100.0	2246	100.0

No significant difference in mean age and BMI was found between the recurrent operations in TEP and TAPP technique (Table 2). That was also true for the ASA-score, defect size, and risk factors (Table 3). With regard to EHS localization, there were significantly more medial recurrent inguinal hernia defects (52.2% vs. 44.8%;* p* < 0.001) and significantly fewer lateral defects (55.4% vs. 68.8%;* p* < 0.001) in the TAPP group (Table 3).

**Table 2:** Age and BMI of patients with laparo-endoscopic recurrent unilateral inguinal hernia repair in men

		TEP	TAPP	*p*
Age [years]	Mean ± STD	58.3 ± 15.8	59.2 ± 15.4	0.186
BMI	Mean ± STD	26.1 ± 3.5	25.9 ± 3.3	0.152

**Table 3:** Demographic- and surgery-related parameters and risk factors of patients with laparo-endoscopic recurrent unilateral inguinal hernia repair in men

			TEP		TAPP		*p*
			n	%	n	%	
ASA-score		I	186	23.79	376	25.63	0.467
		II	454	58.06	849	57.87	
		III/IV	142	18.16	242	16.50	
Defect size		I (< 1,5 cm)	137	17.52	282	19.22	0.524
		II (1,5–3 cm)	510	65.22	950	64.76	
		III (>3 cm)	135	17.26	235	16.02	
Risk factors	Total	yes	249	31.84	438	29.86	0.330
		no	533	68.16	1029	70.14	
	COPD	yes	63	8.06	88	6.00	0.063
		no	719	91.94	1379	94.00	
	Diabetes	yes	45	5.75	84	5.73	0.978
		no	737	94.25	1383	94.27	
	Aortic aneurysm	yes	6	0.77	10	0.68	0.818
		no	776	99.23	1457	99.32	
	Immunosuppression	yes	5	0.64	9	0.61	0.941
		no	777	99.36	1458	99.39	
	Corticoid	yes	9	1.15	11	0.75	0.335
		no	773	98.85	1456	99.25	
	Smoking	yes	88	11.25	174	11.86	0.669
		no	694	88.75	1293	88.14	
	Coagulopathy	yes	11	1.41	22	1.50	0.861
		no	771	98.59	1445	98.50	
	Antiplatelet medication	yes	73	9.34	129	8.79	0.669
		no	709	90.66	1338	91.21	
	Anticoagulation therapy	yes	16	2.05	28	1.91	0.823
		no	766	97.95	1439	98.09	
EHS-classification medial		yes	350	44.76	765	52.15	<**0.001**
		no	432	55.24	702	47.85	
EHS-classification lateral		yes	538	68.80	813	55.42	<**0.001**
		no	244	31.20	654	44.58	
EHS-classification femoral		yes	22	2.81	55	3.75	0.245
		no	760	97.19	1412	96.25	
EHS-classification scrotal		yes	6	0.77	21	1.43	0.168
		no	776	99.23	1446	98.57	

As regards the target parameters, no differences were found between TEP and TAPP recurrent inguinal hernia repair in the intraoperative complications (Table 4). For the postoperative complications, significantly lower values (1.7% vs. 4.6%;* p* < 0.001) were identified for TEP, but that was mainly due to the difference in the seroma rate (0.5% vs. 3.2%;* p* < 0.001) (Table 4).

**Table 4:** Univariable analysis of intra- and postoperative complications, complication-related reoperations, and 1-year follow-up results of patients with laparo-endoscopic recurrent unilateral inguinal hernia repair in men

				TEP		TAPP		*p*
				*n*	%	*n*	%	
Intraoperative complication	Total		yes	13	1.66	13	0.89	0.101
			no	769	98.34	1454	99.11	
	Bleeding		yes	7	0.90	8	0.55	0.332
			no	775	99.10	1459	99.45	
	Injury	Total	yes	8	1.02	9	0.61	0.286
			no	774	98.98	1458	99.39	
		Vascular	yes	4	0.51	4	0.27	0.365
			no	778	99.49	1463	99.73	
		Bowel	yes	0	0.00	5	0.34	0.102
			no	782	100.0	1462	99.66	
		Bladder	yes	2	0.26	0	0.00	0.053
			no	780	99.74	1467	100.0	
Postoperative complication	Total		yes	13	1.66	67	4.57	<**0.001**
			no	769	98.34	1400	95.43	
	Bleeding		yes	10	1.28	19	1.30	0.974
			no	772	98.72	1448	98.70	
	Seroma		yes	4	0.51	47	3.20	<**0.001**
			no	778	99.49	1420	96.80	
	Infection		yes	0	0.00	1	0.07	0.465
			no	782	100.0	1466	99.93	
	Bowel injury		yes	0	0.00	1	0.07	0.465
			no	782	100.0	1466	99.93	
	Wound healing disorders		yes	0	0.00	1	0.07	0.465
			no	782	100.0	1466	99.93	
Reoperation			yes	11	1.41	16	1.09	0.512
			no	771	98.59	1451	98.91	
Recurrence on follow-up			yes	7	0.90	21	1.43	0.275
			no	775	99.10	1446	98.57	
Pain in rest on follow-up			yes	54	6.91	79	5.39	0.146
			no	728	93.09	1388	94.61	
Pain on exertion on follow-up			yes	92	11.76	158	10.77	0.475
			no	690	88.24	1309	89.23	
Pain requiring treatment on follow-up			yes	35	4.48	50	3.41	0.206
			no	747	95.52	1417	96.59	

However, since this did not result in any difference in the complication-related reoperation rate, it only meant that TAPP was associated with a higher seroma rate, which responded to conservative treatment.

On one-year follow-up no differences were detected in the re-recurrence rate after recurrent inguinal hernia repair following TAPP and TEP, or in the rates of pain at rest, pain on exertion, or chronic pain requiring treatment (Table 4).

### Multivariable analyses

For the intraoperative complications, complication-related re-reoperations, and recurrences on follow-up it was not possible to calculate a valid model since the number of positive cases was too small.

### Postoperative complications

The results of the model that explored how the variables related to patient and operation characteristics (surgical technique, previous open primary operation, age, BMI, ASA-score, defect size, and defect localization as well as the presence of risk factors) impacted onset of postoperative complications are illustrated in Table 5 (model matching:* p* < 0.001).

**Table 5:** Multivariable analysis of postoperative complications

Parameter	p value	Category	OR estimate	95% CI	
Procedure	<0.001	TAPP vs TEP	3.010	1.636	5.538
Previous operation	0.044	Suture vs open mesh	1.753	1.016	3.025
EHS-classification medial	0.049	yes vs no	0.457	0.209	0.997
Defect size	0.055	II (1,5–3 cm) vs I (<1,5 cm)	1.052	0.539	2.055
		III (>3 cm) vs I (<1,5 cm)	2.012	0.935	4.329
Risk factors	0.165	yes vs no	1.428	0.864	2.360
Age [10-years-OR]	0.270		1.107	0.924	1.326
EHS-classification femoral	0.297	yes vs no	1.717	0.621	4.746
BMI [5-points-OR]	0.394		0.857	0.601	1.223
ASA-score	0.414	II vs I	0.896	0.473	1.695
		III/IV vs I	1.340	0.592	3.034
EHS-classification scrotal	0.552	yes vs no	1.500	0.394	5.715
EHS-classification lateral	0.646	yes vs no	0.827	0.368	1.860

The postoperative complications, primarily seromas, were affected by the surgical technique. Conduct of TAPP operation (OR = 3.010 [1.636; 5.538];* p* < 0.001) increased the risk of postoperative complications. With a prevalence of 3.6%, this would amount to 53 postoperative complications for every 1000 patients undergoing TAPP operation compared with 18 complications for patients operated on in TEP technique. The risk for development of postoperative complications was also increased in patients with previous open suture repair (OR = 1,753 [1.016; 3.025];* p* = 0.044) and decreased in EHS medial classification (OR = 0.457. [0.209; 0.997].

### Pain at rest

The results of multivariable analysis of pain at rest are presented in Table 6 (model matching: *p* = 0.009). Here BMI was the only influence factor identified (*p* < 0.001). A five-point higher BMI increased pain at rest (5-point OR = 1.483 [1.176; 1.170], but there was no evidence of the surgical technique or previous open primary repair having impacted pain at rest.

**Table 6:** Multivariable analysis of pain at rest in 1-year follow-up

Parameter	p value	Category	OR estimate	95%-CI	
BMI [5-points-OR]	<0.001		1.483	1.176	1.870
Defect size	0.065	II (1,5–3 cm) vs I (<1,5 cm)	0.786	0.511	1.210
		III (>3 cm) vs I (<1,5 cm)	0.444	0.225	0.877
Procedure	0.124	TAPP vs TEP	0.751	0.521	1.082
Previous operation	0.232	Suture vs open mesh	0.798	0.551	1.155
EHS-classification lateral	0.248	yes vs no	0.692	0.370	1.292
Risk factors	0.328	yes vs no	1.222	0.818	1.827
EHS-classification medial	0.372	yes vs no	0.758	0.412	1.394
Age [10-years-OR]	0.513		0.957	0.837	1.093
EHS-classification femoral	0.869	yes vs no	1.081	0.431	2.709
ASA-score	0.899	II vs I	0.941	0.595	1.490
		III/IV vs I	0.851	0.427	1.694
EHS-classification scrotal	0.980	yes vs no	0.000	0.000	I

### Pain on exertion

The results of multivariable analysis of pain on exertion are shown in Table 7 (model matching:* p* < 0.001). These were significantly influenced by age, BMI, and defect size. A higher age (10-year OR = 0.846 [0.767; 0.933]; *p* < 0.001) as well as larger hernias (II vs I: OR = 0.699 [0.508; 0.964]; III vs I: OR = 0.517 [0.318; 0.840];* p* = 0.018) reduced the risk of pain on exertion. Conversely, a five-point higher BMI (5-point OR = 1.289 [1.073; 1.549];* p* = 0.007) increased the pain risk, but there was no evidence of the surgical technique or previous open primary repair having impacted the postoperative complication rate.

**Table 7:** Multivariable analysis of pain on exertion in 1-year follow-up

Parameter	p value	Category	OR estimate	95%-CI	
Age [10-years-OR]	<0.001		0.846	0.767	0.933
BMI [5-points-OR]	0.007		1.289	1.073	1.549
Defect size	0.018	II (1,5–3 cm) vs I (<1,5 cm)	0.699	0.508	0.964
		III (>3 cm) vs I (<1,5 cm)	0.517	0.318	0.840
EHS-classification lateral	0.066	yes vs no	0.646	0.406	1.029
EHS-classification scrotal	0.295	yes vs no	0.338	0.044	2.577
Procedure	0.365	TAPP vs TEP	0.878	0.664	1.163
EHS-classification medial	0.405	yes vs no	0.823	0.520	1.302
ASA-score	0.471	II vs I	0.870	0.623	1.217
		III/IV vs I	1.075	0.646	1.787
Risk factors	0.632	yes vs no	0.925	0.674	1.270
EHS-classification femoral	0.797	yes vs no	1.096	0.543	2.214
Previous operation	0.826	Suture vs open with mesh	0.968	0.727	1.290

### Chronic pain requiring treatment

The results of multivariable analysis of chronic pain requiring treatment are presented in Table 8 (model matching:* p* = 0.020). Here BMI was the only significant influence factor identified (*p* = 0.006). Accordingly, a five-point higher BMI increased the rate of chronic pain requiring treatment (5-point OR = 1.477 [1.121; 1.948]), but there was no evidence of the surgical technique or previous open primary repair having impacted the rate of chronic pain requiring treatment.

**Table 8:** Multivariable analysis of chronic pain requiring treatment in 1-year follow-up

Parameter	p-value	Category	OR estimate	95%-CI	
BMI [5-points-OR]	0.006		1.477	1.121	1.948
Previous operation	0.076	Suture vs open mesh	0.666	0.425	1.043
EHS-classification medial	0.088	yes vs no	0.501	0.227	1.108
Procedure	0.195	TAPP vs TEP	0.742	0.473	1.165
EHS-classification lateral	0.246	yes vs no	0.615	0.270	1.399
Defect size	0.287	II (1,5–3 cm) vs I (<1,5 cm)	0.836	0.486	1.438
		III (>3 cm) vs I (<1,5 cm)	0.515	0.225	1.179
EHS-classification femoral	0.382	yes vs no	1.556	0.578	4.190
Age [10-years-OR]	0.446		0.937	0.793	1.107
Risk factors	0.544	yes vs no	1.166	0.710	1.915
ASA-score	0.842	II vs I	1.048	0.584	1.881
		III/IV vs I	1.252	0.547	2.865
EHS-classification scrotal	0.983	yes vs no	0.000	0.000	I

## Discussion

In the Guidelines of the European Hernia Society (EHS) and the International Endohernia Society (IEHS), TEP and TAPP are recommended as equivalent procedures for recurrent hernia repair following the previous open mesh and suture repair of primary inguinal hernias (1, 10, 18). To date, no prospective randomized trials have been conducted to compare TEP and TAPP for recurrent inguinal hernia repair following previous open primary repair. A Swiss registry study that compared laparo-endoscopic recurrent hernia operations identified a significantly higher intraoperative complication rate and longer operative time for TEP operations, which were much more common than TAPP procedures in the patient group analyzed (19). The postoperative length of hospital stay was longer for patients undergoing TAPP (19). Surgical postoperative complications, general postoperative complications, and conversion rates were not significantly different (19). The authors concluded that the absolute outcome differences are small and that both techniques appear to be safe and effective for patients undergoing laparo-endoscopic repair for unilateral recurrent inguinal hernia (19).

Likewise, the present analysis of data from the Herniamed Registry, in which the proportion of TAPP operations was higher than that of the TEP operations, revealed similar outcomes for the laparo-endoscopic recurrent operations following previous open primary operation. Based on the Herniamed Registry data, no significant differences were found between the recurrent operations in TEP vs TAPP technique with regard to the intraoperative complications, complication-related reoperations, re-recurrence rates, rates of pain at rest, pain on exertion, or chronic pain requiring treatment. Unfavorable results were identified only with regard to the higher seroma rates associated with TAPP; these responded to conservative treatment. The influence variables identified here on multivariable analysis were, in addition to the surgical technique, the previous open primary operation and the EHS-classification medial. A previous open primary suture repair has a higher risk for development of a postoperative complication as a previous open primary mesh repair and the EHS-classification medial a lower risk. The results of multivariable analysis of the other parameters did not find any evidence of any impact exerted by the surgical technique.

Accordingly, this analysis of data from the Herniamed Registry corroborates the findings of the Swiss registry study. Similarly, the Herniamed Registry did not detect any significant differences between TEP and TAPP for recurrent unilateral inguinal hernia repair in men following previous open suture or mesh primary operation. That was true for the intraoperative complications, complication-related reoperations, re-recurrence, pain at rest, pain on exertion, and chronic pain requiring treatment on one-year follow-up. The only difference was that TAPP was associated with a higher seroma rate, which responded to conservative treatment. There was no difference in the other postoperative complications between TEP and TAPP for recurrent repair.

In summary, both TEP and TAPP can be recommended as effective techniques for treatment of recurrent inguinal hernia following previous open primary operation. The decision to use one or the other technique should be based solely on the surgeon’s expertise. The registry study presented here thus confirms the recommendations in the guidelines on laparo-endoscopic treatment of recurrent inguinal hernia following previous open primary operation.

## Herniamed Study Group

### Scientific Board

**Köckerling**, Ferdinand (Chairman) (Berlin); **Bittner**, Reinhard (Rottenburg); **Fortelny**, René (Wien); **Jacob**, Dietmar (Berlin); **Koch**, Andreas (Cottbus); **Kraft**, Barbara (Stuttgart); **Kuthe**, Andreas (Hannover); **Lippert**, Hans (Magdeburg):**Lorenz**, Ralph (Berlin); **Mayer**, Franz (Salzburg); **Moesta**, Kurt Thomas (Hannover); **Niebuhr**, Henning (Hamburg); **Peiper**, Christian (Hamm); **Pross**, Matthias (Berlin); **Reinpold**, Wolfgang (Hamburg); **Simon**, Thomas (Weinheim); **Stechemesser**, Bernd (Köln); **Unger**, Solveig (Chemnitz).

### Participants

Ahmetov, Azat (Saint-Petersburg); **Alapatt**, Terence Francis (Frankfurt/Main); **Albayrak**, Nurettin (Herne); **Amann**, Stefan (Neuendettelsau); **Anders**, Stefan (Berlin); **Anderson**, Jürina (Würzburg); **Antoine**, Dirk (Leverkusen); **Arndt**, Anatoli (Elmshorn); **Asperger**, Walter (Halle); **Avram**, Iulian (Saarbrücken); **Baikoglu-Endres**, Corc (Weißenburg i. Bay.); **Bandowsky**, Boris (Damme); **Barkus;** Jörg (Velbert); **Becker**, Matthias (Freital); **Behrend**, Matthias (Deggendorf); **Beuleke**, Andrea (Burgwedel); **Berger**, Dieter (Baden–Baden); **Birk**, Dieter (Bietigheim-Bissingen); **Bittner**, Reinhard (Rottenburg); **Blaha**, Pavel (Zwiesel); **Blumberg**, Claus (Lübeck); **Böckmann**, Ulrich (Papenburg); **Böhle**, Arnd Steffen (Bremen); **Bolle**, Ludger (Berlin); **Borchert**, Erika (Grevenbroich); **Born**, Henry (Leipzig); **Brabender**, Jan (Köln); **Breitenbuch von**, Philipp (Radebeul); **Brož**, Miroslav (Ebersbach); **Brütting**, Alfred (Erlangen); **Buchert**, Annette (Mallersdorf-Pfaffenberg); **Budzier**, Eckhard (Meldorf); **Burchett**, Bert (Waren); **Burghardt**, Jens (Rüdersdorf); **Cejnar**, Stephan-Alexander (München); **Chirikov**, Ruslan (Dorsten); **Claußnitzer**, Christian (Ulm); **Comman**, Andreas (Bogen); **Crescent**i, Fabio (Verden/Aller); **Daniels**, Thies (Hamburg); **Dapunt**, Emanuela (Bruneck); **Decker**, Georg (Berlin); **Demmel**, Michael (Arnsberg); **Descloux**, Alexandre (Baden); **Deusch**, Klaus-Peter (Wiesbaden); **Dick**, Marcus (Neumünster); **Dieterich**, Klaus (Ditzingen); **Dietz**, Harald (Landshut); **Dittmann**, Michael (Northeim); **Drummer**, Bernhard (Forchheim); **Eckermann**, Oliver (Luckenwalde); **Eckhoff**, Jörn /Hamburg); **Ehmann**, Frank (Grünstadt); **Eisenkrein**, Alexander (Düren); **Elger**, Karlheinz (Germersheim); **Engelhardt**, Thomas (Erfurt); **Erichsen**, Axel (Friedrichshafen); **Eucker**, Dietmar (Bruderholz); **Fackeldey**, Volker (Kitzingen); **Farke**, Stefan (Delmenhorst); **Faust**, Hendrik (Emden); **Federmann**, Georg (Seehausen); **Feichter**, Albert (Wien); **Fiedler**, Michael (Eisenberg); **Fikatas**, Panagiotis (Berlin); **Firl**, Michaela (Perleberg); **Fischer**, Ines (Wiener Neustadt); **Fleischer**, Sabine (Dinslaken); **Fortelny**, René H. (Wien); **Franczak**, Andreas (Wien); **Franke**, Claus (Düsseldorf); **Frankenberg von**, Moritz (Salem); **Frehner**, Wolfgang (Ottobeuren); **Friedhoff**, Klaus (Andernach); **Friedrich**, Jürgen (Essen); **Frings**, Wolfram (Bonn); **Fritsche**, Ralf (Darmstadt); **Frommhold**, Klaus (Coesfeld); **Frunder**, Albrecht (Tübingen); **Fuhrer**, Günther (Reutlingen); **Gassler**, Harald (Villach); **Gawad**, Karim A. Frankfurt/Main); **Gehrig**, Tobias (Sinsheim); **Gerdes**, Martin (Ostercappeln); **Germanov**, German (Halberstadt; **Gilg**, Kai-Uwe (Hartmannsdorf); **Glaubitz**, Martin (Neumünster); **Glauner-Goldschmidt**, Kerstin (Werne); **Glutig**, Holger (Meissen); **Gmeiner**, Dietmar (Bad Dürrnberg); **Göring**, Herbert (München); **Grebe**, Werner (Rheda-Wiedenbrück); **Grothe**, Dirk (Melle); **Gürtler**, Thomas (Zürich); **Hache**, Helmer (Löbau); **Hämmerle**, Alexander (Bad Pyrmont); **Haffner**, Eugen (Hamm); **Hain**, Hans-Jürgen (Gross-Umstadt); **Hammans**, Sebastian (Lingen); **Hampe**, Carsten (Garbsen); **Hanke**, Stefan (Halle); **Harrer**, Petra (Starnberg); **Hartung**, Peter (Werne); **Heinzmann**, Bernd (Magdeburg); **Heise**, Joachim Wilfried (Stolberg); **Heitland**, Tim (München); **Helbling**, Christian (Rapperswil); **Hempen**, Hans-Günther (Cloppenburg); **Henneking**, Klaus-Wilhelm (Bayreuth); **Hennes**, Norbert (Duisburg); **Hermes**, Wolfgang (Weyhe); **Herrgesell**, Holger (Berlin); **Herzing**, Holger Höchstadt); **Hessler**, Christian (Bingen); **Heuer**, Matthias (Herten); **Hildebrand**, Christiaan (Langenfeld); **Höferlin**, Andreas (Mainz); **Hoffmann**, Henry (Basel); **Hoffmann**, Michael (Kassel); **Hofmann**, Eva M. (Frankfurt/Main); **Hornung**, Frederic (Wolfratshausen); **Hügel**, Omar (Hannover); **Hüttemann**, Martin (Oberhausen); **Hunkeler**, Rolf (Zürich); **Imdahl**, Andreas (Heidenheim); **Isemer**, Friedrich-Eckart (Wiesbaden); **Jablonski**, Herbert Gustav (Sögel); **Jacob**, Dietmar (Berlin); **Jansen-Winkeln**, Boris (Leipzig); **Jantschulev**, Methodi (Waren); **Jenert**, Burghard (Lichtenstein); **Jugenheimer**, Michael (Herrenberg); **Junger**, Marc (München); **Kaaden**, Stephan (Neustadt am Rübenberge); **Käs**, Stephan (Weiden); **Kahraman**, Orhan (Hamburg); **Kaiser**, Christian (Westerstede); **Kaiser**, Gernot Maximilian (Kamp-Lintfort); **Kaiser**, Stefan (Kleinmachnow); **Kapischke**, Matthias (Hamburg); **Karch**, Matthias (Eichstätt); **Kasparek**, Michael S. (München); **Keck**, Heinrich (Wolfenbüttel); **Keller**, Hans W. (Bonn); **Kienzle**, Ulrich (Karlsruhe); **Kipfmüller**, Brigitte (Köthen); **Kirsch**, Ulrike (Oranienburg); **Klammer**, Frank (Ahlen); **Klatt**, Richard (Hagen); **Klein**, Karl-Hermann (Burbach); **Kleist**, Sven (Berlin); **Klobusicky**, Pavol (Bad Kissingen); **Kneifel**, Thomas (Datteln); **Knoop**, Michael (Frankfurt/Oder); **Knotter**, Bianca (Mannheim); **Koch**, Andreas (Cottbus); **Koch**, Andreas (Münster); **Köckerling**, Ferdinand (Berlin); **Köhler**, Gernot (Linz); **König**, Oliver (Buchholz); **Kornblum**, Hans (Tübingen); **Krämer**, Dirk (Bad Zwischenahn); **Kraft**, Barbara (Stuttgart); **Kratsch**, Barthel (Dierdorf/Selters); **Kreissl**, Peter (Ebersberg); **Krones**, Carsten Johannes (Aachen); **Kronhardt**, Heinrich (Neustadt am Rübenberge); **Kruse**, Christinan (Aschaffenburg); **Kube**, Rainer (Cottbus); **Kühlberg**, Thomas (Berlin); **Kühn**, Gert (Freiberg); **Kuhn**, Roger (Gifhorn); **Kusch**, Eduard (Gütersloh); **Kuthe**, Andreas (Hannover); **Ladberg**, Ralf (Bremen); **Ladra**, Jürgen (Düren); **Lahr-Eigen**, Rolf (Potsdam); **Lainka**, Martin (Wattenscheid); **Lammers**, Bernhard J. (Neuss); **Lancee**, Steffen (Alsfeld); **Lange**, Claas (Berlin); **Langer**, Claus (Göttingen); **Laps**, Rainer (Ehringshausen); **Larusson**, Hannes Jon (Pinneberg); **Lauschke**, Holger (Duisburg); **Leher**, Markus (Schärding); **Leidl**, Stefan (Waidhofen/Ybbs); **Lenz**, Stefan (Berlin); **Liedke**, Marc Olaf (Heide); **Lienert**, Mark (Duisburg); **Limberger**, Andreas (Schrobenhausen); **Limmer**, Stefan (Würzburg); **Locher**, Martin (Kiel); **Loghmanieh**, Siawasch (Viersen); **Lorenz**, Ralph (Berlin); **Luther**, Stefan (Wipperfürth); **Luyken**, Walter (Sulzbach-Rosenberg); **Mallmann**, Bernhard (Krefeld); **Manger**, Regina (Schwabmünchen); **Maurer**, Stephan (Münster); **May**, Jens Peter (Schönebeck); **Mayer**, Franz (Salzburg); **Mayer**, Jens (Schwäbisch Gmünd); **Mellert**, Joachim (Höxter); **Menzel**, Ingo (Weimar); **Meurer**, Kirsten (Bochum); **Meyer**, Moritz (Ahaus**); Mirow**, Lutz (Kirchberg); **Mittag-Bonsch**, Martina (Crailsheim); **Mittenzwey**, Hans-Joachim (Berlin); **Möbius**, Ekkehard (Braunschweig); **Mörder-Köttgen**, Anja (Freiburg); **Moesta**, Kurt Thomas (Hannover); **Moldenhauer**, Ingolf (Braunschweig); **Morkramer**, Rolf (Xanten); **Mosa**, Tawfik (Merseburg); **Müller**, Hannes (Schlanders); **Münzberg**, Gregor (Berlin); **Murr**, Alfons (Vilshofen); **Mussack**, Thomas (St. Gallen); **Nartschik**, Peter (Quedlinburg); **Nasifoglu**, Bernd (Ehingen); **Neumann**, Jürgen (Haan); **Neumeuer**, Kai (Paderborn); **Niebuhr**, Henning (Hamburg); **Nix**, Carsten (Walsrode); **Nölling**, Anke (Burbach); **Nostitz**, Friedrich Zoltán (Mühlhausen); **Obermaier**, Straubing); **Öz-Schmidt**, Meryem (Hanau); **Oldorf**, Peter (Usingen); **Olivieri**, Manuel (Pforzheim); **Passon**, Marius (Freudenberg); **Pawelzik**, Marek (Hamburg); **Pein**, Tobias (Hameln); **Peiper**, Christian (Hamm); **Peiper**, Matthias (Essen); **Peitgen**, Klaus (Bottrop); **Pertl**, Alexander (Spittal/Drau); **Philipp**, Mark (Rostock); **Pickart**, Lutz (Bad Langensalza); **Pizzera**, Christian (Graz); **Pöllath**, Martin (Sulzbach-Rosenberg); **Possin**, Ulrich (Laatzen); **Prenzel**, Klaus (Bad Neuenahr-Ahrweiler); **Pröve**, Florian (Goslar); **Pronnet**, Thomas (Fürstenfeldbruck); **Pross**, Matthias (Berlin); **Puff**, Johannes (Dinkelsbühl); **Rabl**, Anton (Passau); **Raggi**, Matthias Claudius (Stuttgart); **Rapp**, Martin (Neunkirchen); **Reck**, Thomas (Püttlingen); **Reinpold**, Wolfgang (Hamburg); **Reuter**, Christoph (Quakenbrück); **Richter**, Jörg (Winnenden); **Riemann**, Kerstin (Alzenau-Wasserlos); **Riesener**, Klaus-Peter (Marl); **Rodehorst**, Anette (Otterndorf); **Roehr**, Thomas (Rödental); **Rössler**, Michael (Rüdesheim am Rhein); **Roncossek**, Bremerhaven); **Rosniatowski**, Rolland (Marburg); **Roth** Hartmut (Nürnberg); **Sardoschau**, Nihad (Saarbrücken); **Sauer**, Gottfried (Rüsselsheim); **Sauer**, Jörg (Arnsberg); **Seekamp**, Axel (Freiburg); **Seelig**, Matthias (Bad Soden); **Seidel**, Hanka (Eschweiler); **Seiler**, Christoph Michael (Warendorf); **Seltmann**, Cornelia (Hachenburg); **Senkal**, Metin (Witten); **Shamiyeh**, Andreas (Linz); **Shang**, Edward (München); **Siemssen**, Björn (Berlin); **Sievers**, Dörte (Hamburg); **Silbernik**, Daniel (Bonn); **Simon**, Thomas (Weinheim); **Sinn**, Daniel (Olpe); **Sinner**, Guy (Merzig); **Sinning**, Frank (Nürnberg); **Smaxwil**, Constatin Aurel (Stuttgart); **Sörensen**, Björn (Lauf an der Pegnitz); **Syga**, Günter (Bayreuth); **Schabel**, Volker (Kirchheim/Teck); **Schadd**, Peter (Euskirchen); **Schassen von**, Christian (Hamburg); **Scheidbach**, Hubert (Neustadt/Saale); **Schelp**, Lothar (Wuppertal); **Scherf**, Alexander (Pforzheim); **Scheuerlein**, Hubert (Paderborn); **Scheyer**, Mathias (Bludenz); **Schilling**, André (Kamen); **Schimmelpenning**, Hendrik (Neustadt in Holstein); **Schinkel**, Svenja (Kempten); **Schmid**, Michael (Gera); **Schmid**, Thomas (Innsbruck); **Schmidt**, Ulf (Mechernich); **Schmitz**, Heiner (Jena); **Schmitz**, Ronald (Altenburg); **Schöche**, Jan (Borna); **Schoenen**, Detlef (Schwandorf); **Schrittwieser**, Rudolf /Bruck an der Mur); **Schroll**, Andreas (München); **Schubert**, Daniel (Saarbrücken); **Schüder**, Gerhard (Wertheim); **Schultz**, Christian (Bremen-Lesum); **Schultz**, Harald (Landstuhl); **Schulze**, Frank P. Mülheim an der Ruhr); **Schulze**, Thomas (Dessau-Roßlau); **Schumacher**, Franz-Josef (Oberhausen); **Schwab**, Robert (Koblenz); **Schwandner**, Thilo (Lich); **Schwarz**, Jochen Günter (Rottenburg); **Schymatzek**, Ulrich (Eitorf); **Spangenberger**, Wolfgang (Bergisch-Gladbach); **Sperling**, Peter (Montabaur); **Staade**, Katja (Düsseldorf); **Staib**, Ludger (Esslingen); **Staikov**, Plamen (Frankfurt am Main); **Stamm**, Ingrid (Heppenheim); **Stark**, Wolfgang (Roth); **Stechemesser**, Bernd (Köln); **Steinhilper**, Uz (München); **Stengl**, Wolfgang (Nürnberg); **Stern**, Oliver (Hamburg); **Stöltzing**, Oliver (Meißen); **Stolte**, Thomas (Mannheim); **Stopinski**, Jürgen (Schwalmstadt); **Stratmann**, Gerald (Goch); **Stubbe**, Hendrik (Güstrow/); **Stülzebach**, Carsten (Friedrichroda); **Tepel**, Jürgen (Osnabrück); **Terzić**, Alexander (Wildeshausen); **Teske**, Ulrich (Essen); **Tichomirow**, Alexej (Brühl); **Tillenburg**, Wolfgang (Marktheidenfeld); **Timmermann**, Wolfgang (Hagen); **Tomov**, Tsvetomir (Koblenz; **Train**, Stefan H. (Gronau); **Trauzettel**, Uwe (Plettenberg); **Triechelt**, Uwe (Langenhagen); **Ulbricht**, Wolfgang (Breitenbrunn); **Ulcar**, Heimo (Schwarzach im Pongau); **Unger**, Solveig (Chemnitz); **Verweel**, Rainer (Hürth); **Vogel**, Ulrike (Berlin); **Voigt**, Rigo (Altenburg); **Voit**, Gerhard (Fürth); **Volkers**, Hans-Uwe (Norden); **Volmer**, Ulla (Berlin); **Vossough**, Alexander (Neuss); **Wallasch**, Andreas (Menden); **Wallner**, Axel (Lüdinghausen); **Warscher**, Manfred (Lienz); **Warwas**, Markus (Bonn); **Weber**, Jörg (Köln); **Weber**, Uwe (Eggenfelden); **Weihrauch**, Thomas (Ilmenau); **Weiß**, Johannes (Schwetzingen); **Weißenbach**, Peter (Neunkirchen); **Werner**, Uwe (Lübbecke-Rahden); **Wessel**, Ina (Duisburg); **Weyhe**, Dirk (Oldenburg); **Wieber**, Isabell (Köln); **Wiesmann**, Aloys (Rheine); **Wiesner**, Ingo (Halle); **Withöft**, Detlef (Neutraubling); **Woehe**, Fritz (Sanderhausen); **Wolf**, Claudio (Neuwied); **Wolkersdörfer**, Toralf (Pößneck); **Yaksan**, Arif (Wermeskirchen); **Yildirim**, Can (Lilienthal); **Yildirim**, Selcuk (Berlin); **Zarras**, Konstantinos (Düsseldorf); **Zeller**, Johannes (Waldshut-Tiengen); **Zhorzel**, Sven (Agatharied); **Zuz**, Gerhard (Leipzig);
